# Harmonising Evidence-based medicine teaching: a study of the outcomes of e-learning in five European countries

**DOI:** 10.1186/1472-6920-8-27

**Published:** 2008-04-29

**Authors:** Regina Kulier, Julie Hadley, Susanne Weinbrenner, Berrit Meyerrose, Tamas Decsi, Andrea R Horvath, Eva Nagy, Jose I Emparanza, Sjors FPJ Coppus, Theodoros N Arvanitis, Amanda Burls, Juan B Cabello, Marcin Kaczor, Gianni Zanrei, Karen Pierer, Katarzyna Stawiarz, Regina Kunz, Ben WJ Mol, Khalid S Khan

**Affiliations:** 1 The University of Birmingham, Edgbaston, Birmingham B15 2TG, UK; 2 Birmingham Women's Hospital, Metchley Park Road, Edgbaston, Birmingham B15 2TG, UK; 3 Agency for Quality in Medicine, Weglelystrasse 3, 10623 Berlin, Germany; 4 University of Pécs, Department of Paediatrics, József Attila u. 7, Pécs, H-7623, Hungary; 5 TUDOR, University of Szeged, Albert Szent-Gyorgyi Medical and Pharmacological Centre, Somogyi Bela ter 1, Szeged, H-6725, Hungary; 6 CASPe (CASP Espana), Joaquin Orozco 6, 1°-F, 03006 Alicante, Spain; 7 Academic Medical Center, University of Amsterdam, Department of Obstetrics and Gynaecology, Meibergdreef 9, 1105 AZ Amsterdam, The Netherlands; 8 Academic Medical Center, University of Amsterdam, Department of Clinical Epidemiology and Biostatistics, Meibergdreef 9, 1105 AZ Amsterdam, Amsterdam, The Netherlands; 9 CASPolska, 30-347 Krakow, ul. Wadowicka 3, Poland; 10 Universitá Cattolica del Sacro Cuore, Via Emilia Parmense 84, 29100 Piacenza, Italy; 11 Basel Institute for Clinical Epidemiology, Hebelstrasse 10, CH 4031 Basel, Switzerland

## Abstract

**Background:**

We developed and evaluated the outcomes of an e-learning course for evidence based medicine (EBM) training in postgraduate medical education in different languages and settings across five European countries.

**Methods:**

We measured changes in knowledge and attitudes with well-developed assessment tools before and after administration of the course. The course consisted of five e-learning modules covering acquisition (formulating a question and search of the literature), appraisal, application and implementation of findings from systematic reviews of therapeutic interventions, each with interactive audio-visual learning materials of 15 to 20 minutes duration. The modules were prepared in English, Spanish, German and Hungarian. The course was delivered to 101 students from different specialties in Germany (psychiatrists), Hungary (mixture of specialties), Spain (general medical practitioners), Switzerland (obstetricians-gynaecologists) and the UK (obstetricians-gynaecologists). We analysed changes in scores across modules and countries.

**Results:**

On average across all countries, knowledge scores significantly improved from pre- to post-course for all five modules (p < 0.001). The improvements in scores were on average 1.87 points (14% of total score) for module 1, 1.81 points (26% of total score) for module 2, 1.9 points (11% of total score) for module 3, 1.9 points (12% of total score) for module 4 and 1.14 points (14% of total score) for module 5. In the country specific analysis, knowledge gain was not significant for module 4 in Spain, Switzerland and the UK, for module 3 in Spain and Switzerland and for module 2 in Spain. Compared to pre-course assessment, after completing the course participants felt more confident that they can assess research evidence and that the healthcare system in their country should have its own programme of research about clinical effectiveness.

**Conclusion:**

E-learning in EBM can be harmonised for effective teaching and learning in different languages, educational settings and clinical specialties, paving the way for development of an international e-EBM course.

## Introduction

E-learning is becoming increasingly popular as a tool to aid teaching and learning in medical education. It has several advantages over traditional face-to-face teaching [1-3]. It allows flexibility, enabling busy clinicians to choose the time and place for learning within their clinical duties. Learning can be timed at an individual's own pace and re-visited whenever necessary. E-learning packages can range from a simple online collection of resources to supplement traditional teaching to a fully web-based interactive course with all teaching materials, assessments and support provided online. How would teaching and learning in evidence-based medicine (EBM) fare if delivered via e-learning?

EBM requires mastery in knowledge acquisition [4,5], and e-learning with live web links to relevant information sources can lead trainees to directly obtain relevant learning experience. Studies comparing e-teaching to traditional teaching methods in undergraduate teaching [1,6-8] show that it has educational advantages but requires training of staff and students. In postgraduate programmes, it has been shown that e-learning EBM leads to equivalent knowledge and attitudinal gains compared to face-to-face lectures [9]. As key knowledge sources for EBM are universally accessible via the Internet, web-based teaching may also allow for standardisation of teaching over larger geographical distances and a range of languages, settings and cultures, helping to achieve harmonisation in certification of competence. The success of this aspect of e-learning has not been empirically examined.

We developed an e-EBM course, translated it into various languages and carried out an evaluation among postgraduate medical trainees across five European countries to evaluate whether such a course was feasible within Europe.

## Methods

We conducted the study in Germany, Hungary, Spain, Switzerland and the UK using a before and after design to examine the effect of e-learning on participants' knowledge and attitudes measured with validated assessment tools [10-12].

### Description of the e-learning course

We developed an e-learning course for teaching EBM in postgraduate trainees [13]. The curriculum was prepared by the EU EBM Unity partnership [14], funded by the European Union's vocational training programme (Leonardo da Vinci). The EU EBM Unity, a collaborative pilot project involving 11 partners within Europe, aims to contribute to harmonisation of EBM teaching across the European healthcare sector. Using an established methodology of curriculum development [15] we defined explicit learning objectives about knowledge, skills, attitudes and behaviour for five teaching modules covering the various EBM steps shown in table 1. In brief, the modules cover the following steps: module 1: asking clinical questions, module 2: searching the evidence, module 3: critical appraisal of systematic reviews, module 4: applicability of the evidence to the patient and module 5: implementation of evidence into practice.

**Table 1:** An overview of e-EBM course curriculum

**Aim**: To familiarise course participants with evidence based medicine (EBM) basics
**Target participants**: Health professionals in a clinical setting.
**Learning objectives:**
Upon the completion of the course, participants should be competently able to:
• generate structured questions arising from clinical problems in practice
• search relevant literature, identifying systematic reviews wherever possible
• assess the quality (validity) of systematic reviews and primary research included within them
• assess the applicability of research findings in clinical practice
• effectively implement the output from above activities into clinical practice
**E-learning modules:**
Five models provide learning materials
Module 1: Asking clinical questions
Module 2: Searching the evidence
Module 3: Critical appraisal of systematic reviews (and their constituent studies)
Module 4: Applicability of the evidence to the patient
Module 5: Implementation of evidence into practice
**Learning/teaching methods**
• Participants to pursue independent study by using the e-learning modules and to undertake formal assessment
**Assessment**
∘ Multiple choice questions to test knowledge
∘ Questionnaire to test attitudes

**Figure 1:**
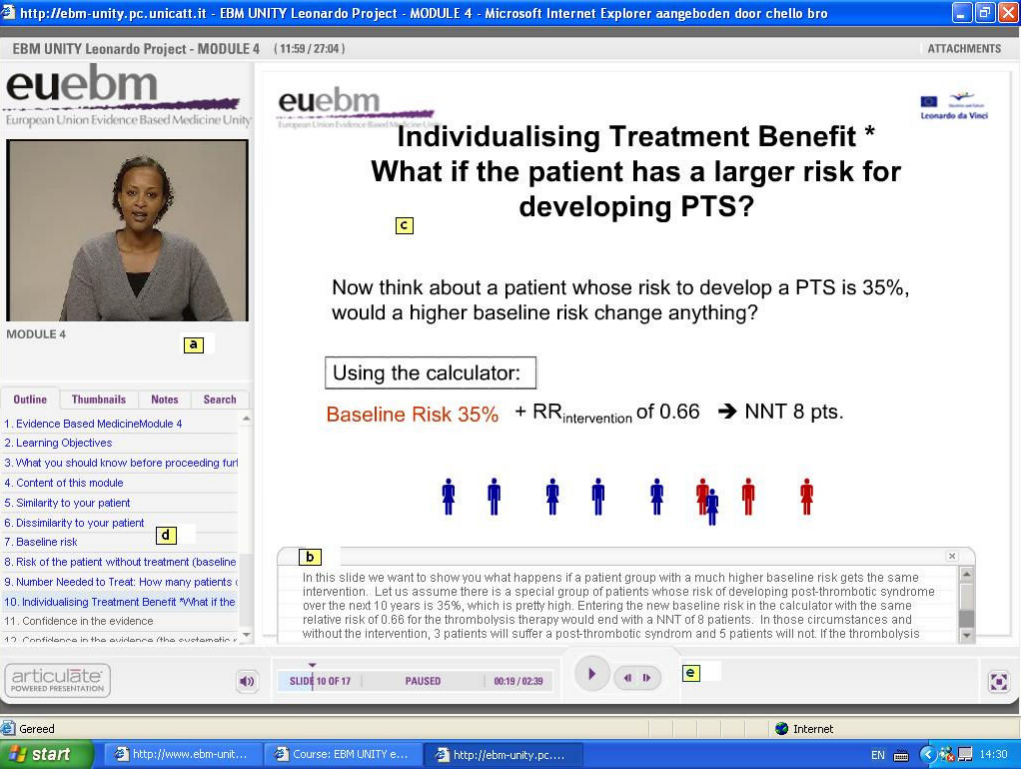
**Online presentation of the e-session**. Screenshot demonstrating various audiovisual teaching modalities that are applied in the e-learning session to support self-directed learning: a) a person is talking to the learner; b) the learner can follow the presentation by reading the notes; c) the slide summarises the core content of the presentation; it may contain hyperlinks to other topics in the same module; d) the sidebar provides orientation to the learner about the content of the lecture; e) the bottom bar allows the learner to pause, or quickly navigate forth and back.

Each module consists of a combination of different teaching and learning methods, including web-based e-learning materials. This study describes the evaluation of the e-learning materials, consisting of e-sessions and assessment. The e-sessions provide the theoretical EBM knowledge for participants to apply in a real clinical situation. The sessions focus on acquisition, appraisal, application and implementation of findings from systematic reviews of therapeutic effectiveness (table 1). E-sessions (figure 1) consist of slides with audio (recorded text) and visual (recorded video and written text) components (Adobe Presenter) with each session taking between 15 to 20 minutes. Each session commences with presenting the learning objectives, followed by the main learning content and ends with verifying that the learning objectives have been covered. The main content incorporates interactive features, flexibility to navigate between subtopics, and links to other modules and relevant websites. The materials can be accessed via the project's website or via CD-ROM [14]. Assessments are carried out at the end of each session. The content of the training materials are for medical postgraduate trainees in general. The systematic review referred to in the examples is about treatment of deep venous thrombosis, a condition known and important to all medical specialties.

E-learning materials were prepared in English and translated into other languages. The extent of translation for each country depended on its educational system and practice. In Switzerland the English version was used as competence in this language was common among the participants. In Spain the instructions and assessments were translated into Spanish, while the English version was used for the e-sessions. In Germany and Hungary all materials were translated into the national language.

### Administration of e-learning courses

We conducted the study between March and July 2007. Before administration of the course, a facilitator explained the nature of the course and the study to all participants who provided verbal consent for use of their responses to assessments. The facilitator was usually the principal investigator in the country and had participated in the development of the materials. Participation to the course was voluntary and participants could leave the study at any point.

At the beginning, participants filled in a pre-course assessment of knowledge and attitudes. The e-sessions were then presented to the participants consecutively (starting with e-session module 1). After each module the participants completed the assessment for that module straight after before moving on to the next module. Any questions from participants were answered by the facilitator after the final session. All modules were administered on a single day in Germany and the UK. In Hungary, Spain and Switzerland modules were completed over 2–3 days, with the pre-course assessment on the first day. Presentation of the modules was via projection and speakers in a lecture hall in Germany, Spain, Switzerland and UK. In Hungary, small groups of participants were listening to the presentation by using a notebook and earphones. Participants completed the attitude questionnaire and a short usability questionnaire after the last session. The aim of the usability questions was to find out about participants' view on the feasibility and quality of the web-based materials. Similarly, facilitators were asked to fill in a short questionnaire about their view on the quality and usefulness of the present course and teaching of EBM in general.

### Outcome measures (Assessments)

**Figure 4:**
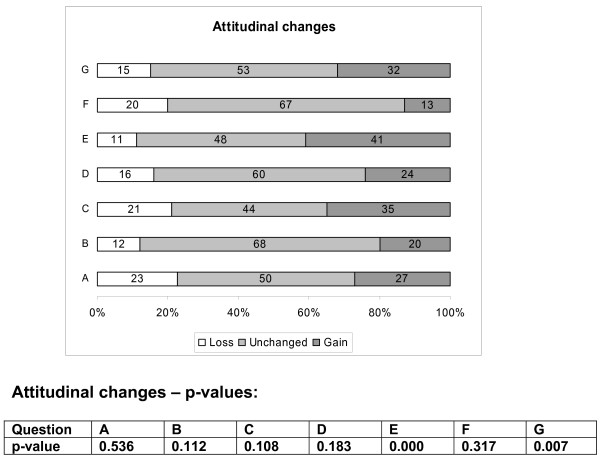
**Attitudinal gains in the e-EBM course (all participants)**. Legend for the questions: (A): Original research is confusing (B) Study design is important in article selection (C) Evidence-based decision making is ' health care by numbers' (D) Contracts for health care professionals should include time taken away from patient care for reading and appraising the literature (E) I am confident that I can assess research evidence (F) Systematic reviews play a key role in informing evidence-based decision making (G) The health care system in my country should have its own programme of research about clinical effectiveness. Attitudinal gains were significant for questions E (p = 0.000) and G (p = 0.007) only; 41% and 32% of participants showed an attitudinal gain in questions E and G respectively (see methods section for details)

We developed a questionnaire to measure knowledge and attitudes. The questions had previously been validated [10-12]. We adapted the knowledge questions to match the learning objectives of this course so as to have content validity. Participants completed the questionnaire before and after the e-sessions as outlined above. The questionnaire contained two types of questions: choice between two answers ('true' or 'false') and 'best fitting answer' (choice of one out of five answers). We used questions adapted from previously validated questionnaires to assess attitude towards EBM [12] (figure 4). Responses were possible on a five point Likert scale ranging from 'strongly agree' to 'strongly disagree' and 'don't know' (the latter was excluded from the analysis). We translated the questionnaires into each partner countries' language in Hungary, Spain and Germany to avoid misunderstandings due to language difficulties. The questionnaires were completed on paper and all data were entered electronically by the investigators at the end of the course.

### Data Analysis

Responses to the knowledge questionnaires of the five modules were scored and pre-course scores were compared to post-course scores using paired Wilcoxon signed ranks tests. We computed the change in knowledge as the difference between scores post-course and scores pre-course. Thus a positive difference meant a gain in knowledge. The number of questions was different per module and the maximum score per module was 13 for module 1, 7 for module 2, 18 for module 3, 16 for module 4 and 8 for module 5. We computed percentage of gain in knowledge using the maximal points that can be achieved in each module as denominator. Data were summarised as percentage mean difference and standard deviation between scores post- course and scores pre-course to obtain a relative measure of the change for every module. We computed attitudinal change either as gain, unchanged or loss, comparing the direction of the answer after the course with the response at baseline. Attitudinal gain was defined as any change (of whatever magnitude), towards a more positive attitude to EBM as measured with the Likert scale. For question A and C (figure 4), an attitudinal gain was observed whenever there was a shift towards the 'strongly disagree' end of the scale. For the rest of the questions the shift was in the opposite direction. An attitudinal loss was defined as a change in the Likert scale against EBM as computed in a way similarly to above. Finally, we coded response as 'unchanged' attitude if pre-course and post-course Likert scores were the same. Attitudinal changes between pre- and post -course Likert scales were compared by means of a Wilcoxon signed ranks test.

## Results

**Figure 2:**
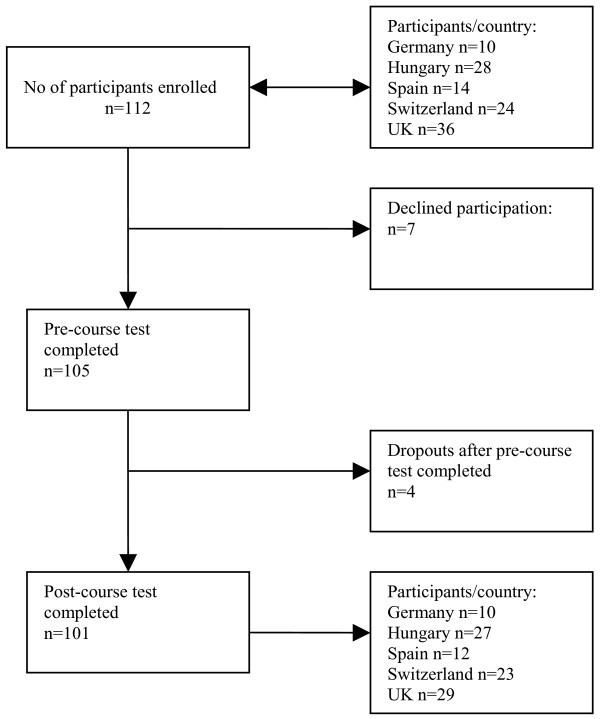
Flowchart of participants in the e-EBM study.

Figure 2 shows the flow of participants in the study. There were 101 complete sets of responses to questionnaires from difference specialties. In the UK local trainee obstetricians-gynaecologists, in Switzerland obstetricians-gynaecologists from low-and middle income countries, in Germany local trainee psychiatrists, in Hungary residents from different specialties and in Spain medical practitioners participated. Age of the participants varied and therefore their level of clinical experience. In Switzerland, for example, participants were aged between 28 to 49 years.

In the UK, only four participants were able to attend the presentation of the e-session for module 4 due to other professional commitments. Almost all participants indicated that they are currently clinically active (92/101). Most participants in the UK (20/29), about half of all participants in Spain (5/12), 7/17 in Switzerland, 3/27 in Hungary and none in Germany had previous formal, structured EBM training.

**Figure 3:**
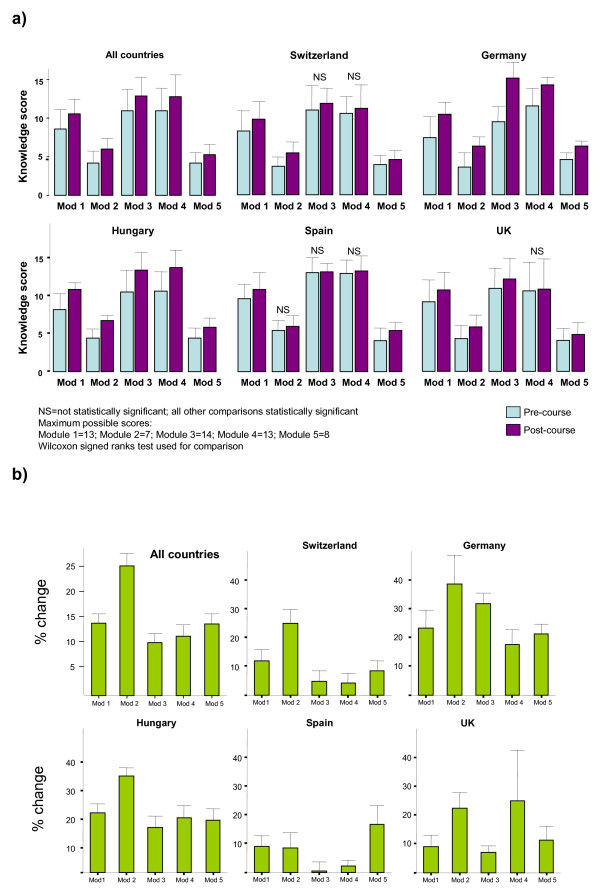
Knowledge gain in the five e-EBM modules in all centres and per country: **(a) **absolute values of pre- and post course scores; **(b) **mean knowledge gain as a percent of total score per module.

In the combined analysis including all centres, participants gained knowledge in all five modules (figure 3 and table 2). The improvements in scores were on average 1.87 points for module 1, 1.81 points for module 2, 1.9 points for module 3, 1.9 points for module 4 and 1.14 points for module 5. In percentages, the participants gained on average 14% of score of the total possible score in module 1, 26% in module 2, 11% in module 3, 12% in module 4 and finally 14% in module 5. All differences in scores between pre-course and post-course questionnaires were statistically significant. This significance was also present in all modules in the country-specific analyses for Germany and Hungary. Knowledge gain was not significant for module 4 in Spain, Switzerland and the UK, for module 3 in Spain and Switzerland and for module 2 in Spain.

**Table 2:** Knowledge gain in the five e-EBM modules in all centres and per country presented as mean difference of pre- post course scores and the mean difference as a percent of total score per module.

**All countries**							
	Number of participants	Average gain in score	SD	p	Average gain in %	SD	p
Module 1	96	1.87	2.41	< 0.001	0.14	0.19	< 0.001
Module 2	100	1.81	1.72	< 0.001	0.26	0.25	< 0.001
Module 3	99	1.90	3.14	< 0.001	0.11	0.17	< 0.001
Module 4	77	1.90	3.19	< 0.001	0.12	0.20	< 0.001
Module 5	93	1.14	1.56	< 0.001	0.14	0.20	< 0.001
**UK**							
Module 1	23	1.17	2.52	0.026	0.09	0.19	0.026
Module 2	27	1.56	2.04	0.001	0.22	0.29	0.001
Module 3	29	1.24	2.31	0.009	0.07	0.13	0.009
Module 4	4	4.00	5.60	0.285	0.25	0.35	0.285
Module 5	20	0.90	1.74	0.049	0.11	0.22	0.049
**Switzerland**							
Module 1	24	1.54	2.54	0.003	0.12	0.20	0.003
Module 2	24	1.75	1.59	0.000	0.25	0.23	0.000
Module 3	21	0.86	2.89	0.117	0.05	0.16	0.117
Module 4	24	0.67	2.71	0.167	0.04	0.17	0.167
Module 5	24	0.67	1.40	0.040	0.08	0.18	0.040
**Germany**							
Module 1	10	3.00	2.62	0.016	0.23	0.20	0.016
Module 2	10	2.70	2.16	0.009	0.39	0.31	0.009
Module 3	10	5.70	2.16	0.005	0.32	0.12	0.005
Module 4	10	2.80	2.53	0.005	0.18	0.16	0.005
Module 5	10	1.70	0.82	0.004	0.21	0.10	0.004
**Hungary**							
Module 1	27	2.67	2.17	< 0.001	0.21	0.17	< 0.001
Module 2	27	2.33	1.07	< 0.001	0.33	0.15	< 0.001
Module 3	27	2.81	3.52	< 0.001	0.16	0.20	< 0.001
Module 4	27	3.04	3.46	< 0.001	0.19	0.22	< 0.001
Module 5	27	1.44	1.63	< 0.001	0.18	0.20	< 0.001
**Spain**							
Module 1	12	1.17	1.64	0.044	0.09	0.13	0.044
Module 2	12	0.58	1.31	0.149	0.08	0.19	0.149
Module 3	12	0.08	1.98	0.683	0.00	0.11	0.683
Module 4	12	0.33	1.07	0.271	0.02	0.07	0.271
Module 5	12	1.33	1.78	0.035	0.17	0.22	0.035

Effect of e-learning on attitudinal change towards EBM showed that a proportion of participants showed a more positive attitude towards EBM (figure 4). Taking statistically significant results into account, compared to pre-course assessment, after completing the course participants felt more confident that they can assess research evidence and that the healthcare system in their country should have its own programme of research about clinical effectiveness.

## Discussion

### Main findings

This study shows that EBM teaching with e-learning materials leads to knowledge and attitudinal gain across different countries, languages and settings. After the course, participants possessed knowledge about acquisition, appraisal and application of findings from systematic reviews. They felt that systematic reviews played a key role in EBM and that healthcare systems should have its own programme of research about clinical effectiveness. Perhaps more importantly, our findings demonstrate that e-learning sessions in EBM can be harmonised for effective teaching and learning across different countries, paving the way for development of an international e-EBM course.

### Strengths and weaknesses

To our knowledge, this is the first evaluation of e-EBM in postgraduate education in different languages, educational settings, medical disciplines and countries. The before-and-after design allowed for us to efficiently pilot the effect of the teaching materials. The absence of a control group can be seen as a shortcoming of this study, but the assessments before the course served as control for each individual. Because the before-and-after evaluations were conducted over a very brief period, the effect of external influences are likely to be negligible and we can be reasonably sure the gain in knowledge was due to the effect of the e-learning course.

Assessors were not blinded towards the pre-course scores of the participants. However, outcome measures were differences between objectively measured scores and it is unlikely that being unblinded to pre-course scores could have influenced the results. The dropout rate was small; reassuring us that computer-based learning is a feasible and acceptable method of learning in postgraduate education.

Although, statistical significant change in knowledge scores was observed between pre-and post-course tests, we cannot assume that the increase in knowledge would continue beyond the testing phase without additional follow-up assessment. To address this void, we have commenced a multi-centre randomised-controlled trial with follow-up assessment to determine if the findings are not only statistically significant but also educationally significant and that the knowledge gained is in fact retained.

In some countries, knowledge gain did not reach statistical significance for certain modules. In the UK this could be due to lack of statistical power because of small sample, in Spain this could be due to a ceiling effect because of high baseline knowledge of the participants. In Switzerland, a heterogeneous linguistic background of the participants may have contributed to poor understanding of the contents presented in the English language. Also, the contents presented in some of the modules may have been inherently difficult to learn in a single session.

Attitudinal gains towards EBM were seen across two aspects. The lack of significance across all facets of attitudes tested could be because of already high pre-course attitudes of course participants, making improvement impossible. However, the areas where improvement was noted were directly related to the course content. We are therefore confident that the promising findings of our study demonstrate the feasibility of harmonising e-EBM effectively across countries.

### Meaning of our findings

The aim of our study was to evaluate the feasibility of a multilingual e-EBM course and we have shown that such a course is likely to be successful in providing EBM training across countries within the EU. We cannot draw conclusions about the effect of the course on behavioural change or long-term educational outcomes. E-learning has inherent flexibility for adaptation of teaching and learning materials across countries [16]. Feedback from participants and facilitators revealed that the quality of the e-EBM materials was good and at an adequate difficulty level. The qualitative feedback we received from participants indicated that most found the course useful, the materials to be of good quality and the difficulty level of the content adequate. However, some indicated that '...*the speaker should be more enthusiastic... *'some sessions are too long: '..*session four is too long, it is difficult to follow..*' Almost all participants welcomed the development of further courses. Facilitators agreed that EBM teaching is essential for clinical practice and found the training materials adequate.

We are, however aware that our study was conducted in a more supervised and controlled environment than may be the case in a real setting, which could influence future results.

### Recommendations for practice

Based on our findings, web-based EBM training can be offered across different countries to improve knowledge and attitudes. The EU EBM partnership aims to evaluate the current project in a randomised controlled trial and expand and adapt it to cover subjects other than systematic reviews of effectiveness. Translation and adaptation in a wider range of languages is needed. These developments can lead towards standardisation of workforce EBM competence across countries.

## Competing interests

The authors declare that they have no competing interests.

## Authors' contributions

RKulier, JH, SC, KK, RKunz, BWM wrote the first draft; SW, BM, TD, AH, EN, JE, JC collected the data in the different countries; GZ performed the recording of the modules. All authors contributed substantially to the development of the curriculum and all authors contributed to the final version of the manuscript.

## Pre-publication history

The pre-publication history for this paper can be accessed here:


